# Automated Driving with Cooperative Perception Based on CVFH and Millimeter-Wave V2I Communications for Safe and Efficient Passing through Intersections

**DOI:** 10.3390/s21175854

**Published:** 2021-08-30

**Authors:** Ryuichi Fukatsu, Kei Sakaguchi

**Affiliations:** Department of Electrical & Electronic Engineering, Tokyo Institute of Technology, Meguro-ku, Tokyo 152-8552, Japan; sakaguchi@mobile.ee.titech.ac.jp

**Keywords:** 5G, automated driving, connected cars, cooperative perception, collective perception, V2I communication, V2X communication, millimeter-wave communication, extended sensor

## Abstract

The development of automated driving is actively progressing, and connected cars are also under development. Connected cars are the technology of connecting vehicles to networks so that connected vehicles can enhance their services. Safety services are among the main services expected in connected car society. Cooperative perception belongs to safety services and improves safety by visualizing blind spots. This visualization is achieved by sharing sensor data via wireless communications. Therefore, the number of visualized blind spots highly depends upon the performance of wireless communications. In this paper, we analyzed the required sensor data rate to be shared for the cooperative perception in order to realize safe and reliable automated driving in an intersection scenario. The required sensor data rate was calculated by the combination of recognition and crossing decisions of an automated driving vehicle to adopt realistic assumptions. In this calculation, CVFH was used to derive tight requirements, and the minimum required braking aims to alleviate the traffic congestion around the intersection. At the end of the paper, we compare the required sensor data rate with the outage data rate realized by conventional and millimeter-wave communications, and show that millimeter-wave communications can support safe crossing at a realistic velocity.

## 1. Introduction

Connecting automated driving vehicles with other devices such as vehicles and roadside units (RSUs) is a key technology to improve the safety of automated driving. The importance of this technology is already understood so the development has been started internationally. In Japan, connected cars that can communicate through a network are expected to support automated driving and are planned to be fully realized by 2030 [1]. In America, the National Highway Traffic Safety Administration (NHTSA) performed the evaluation of safety applications for connected vehicles [2]. In Europe, the European Commission has published the ethics of connected and automated vehicles to tackle ethical issues [3]. Since the technology of connected vehicles can improve driving quality, integrating connected vehicles with automated driving is expected to drastically reduce traffic accidents.

Connected vehicles can expand and develop services for vehicles such as safety services and infotainment services. Among these services, we focused on the cooperative or collective perception which is one of the applications of the safety services to improve the safety of automated driving. Cooperative perception is a technology through which a vehicle can use sensor data obtained from other vehicles or RSUs through wireless communications [4]. The effect of cooperative perception comes from obtaining the sensor data of other perspectives so that the receiving vehicle can see through blind spots. In other words, since dynamic maps used for navigation and avoiding obstacles of automated driving are made from sensor data, cooperative perception can be regarded as integrating dynamic maps, which will lead to a higher quality of automated driving.

There are two ways to realize cooperative perception, i.e., sharing processed or raw sensor data. Processed sensor data include information about recognized objects such as the category and location. The main advantage of sharing processed sensor data is that complex processes such as object recognition can be performed in application servers and the high performance of wireless communications is not required, but latency caused by processes cannot be ignored. On the other hand, when raw sensor data are shared, the received vehicle performs a recognition process in its system so that recognition results are free from errors due to the sender. However, in order to support sharing raw sensor data, large amounts of communication resources must be prepared to deal with the total sensor bandwidth from 3 to 40 Gbps [5]. In order to guarantee safe automated driving by cooperative perception, the shared amount of processed or raw sensor data and the rate of sharing required by safe automated driving must be clarified. For example, in [6], the authors published the requirements for sharing both processed and raw sensor data. On the other hand, in [7], sharing raw sensor data was not considered due to the necessity of a large data rate.

In this paper, sharing raw sensor data is assumed for distributed verification, and the utilization of millimeter-wave communications is considered to tackle the problem of communication resources and to benefit from the advantages of sharing raw sensor data. This paper is the extended work of our previous work and the contribution of this paper consists of two aspects as follows [8,9]:

- The first aspect is the derivation of the required sensor data rate for passing through an intersection safely. Although millimeter-wave communications are believed to play an important role in sharing raw sensor data, the minimum required amount of raw sensor data that is transmitted for safe automated driving via millimeter-wave communications is unclear. Therefore, we focused on the derivation of the minimum required sensor data rate for safe automated driving and included the minimum required braking to alleviate traffic congestion and a practical recognition process to derive realistic requirements.
- The second aspect is showing the ability of millimeter-wave communications to support sharing raw sensor data for safe crossing. Since millimeter-wave communications are expected to support safe automated driving, we will show how safe the automated driving millimeter-wave communications can be.

By comparison with previous works [8,9], we extended the driving scenario from overtaking to crossing an intersection, and adopted a more practical recognition process than using edge points.

The rest of this paper is organized as follows. Section 2 introduces related works about the requirements of cooperative perception and the relation between cooperative perception and communication systems. Section 3 shows the assumed intersection scenario and derives the required sensor data rate to pass through the intersection safely. Section 4 discusses the required sensor data rate with the outage capacity of conventional and millimeter-wave communications. Section 5 concludes this paper.

## 2. Related Works

Since dynamic maps are made from sensor data, receiving sensor data from an RSU can be regarded as receiving local dynamic maps that contain information around the RSU. Local dynamic maps are planned for the safe and successful operation of intelligent transport systems (ITS) applications in European Telecommunications Standards Institute (ETSI) [10]. Local dynamic maps handle location information and four types of information classified by the frequency of change. In this case, local dynamic maps are assumed to be stored in ITS stations so that processed sensor data are used for applications in ITS. For example, the intersection collision risk warning is one of ITS applications [11]. When a roadside ITS detects the collision risk at an intersection, this application sends a decentralized environmental notification message (DENM) to approaching vehicles. On the other hand, in [7], ETSI has studied the collective perception services that can be regarded as sharing local dynamic maps. In this analysis, the transmission of raw sensor data is not considered due to high requirements so that processed data based on radar sensors are assumed in the simulation. The results discuss the relation between the generated channel load resulting from transmitting the information of perceived objects and the awareness generated by sensors. The key point of compatibility between high resolution in terms of object information and reduction in channel load is to include the minimum required information about perceived objects. In [12,13], the authors also studied message generation rules for collective perception services and analyzed the trade-off between channel load and service quality and between performance and efficiency.

Cooperative perception or collective perception itself has also been studied by many researchers. In [14], Shan et al. performed cooperative perception with an intelligent RSU in the real urban traffic environment that has an intersection. The intelligent RSU is equipped with a camera and a LiDAR sensor and the detection result is sent to vehicles in a ETSI collective perception message (CPM) format. The receiving vehicle not only receives the location of the shared perceived objects but also the uncertainty bounds of the objects. In [15], Tsukada et al. developed and conducted a roadside perception unit for automated driving. The developed cooperative perception sends cooperative awareness messages (CAMs) encoded into CPMs to vehicles and the receiving vehicles know the location of the shared perceived objects. In [16], Dhawankar et al. proposed a framework for a cooperative platoon of autonomous vehicles. The cooperative platoon is controlled by sharing periodic safety information such as traffic information under a channel estimation model for V2I communication using IEEE 802.11p. The numerical results show that the purposed framework improves cooperative platoon driving.

Realizing cooperative perception by sending raw sensor data has also been studied. In [17], raw LiDAR sensor data were exchanged through 60 GHz wireless communication, that was one of the millimeter-wave communications. The main characteristics of this work are that only point cloud data representing dynamic objects are shared in order to reduce redundant information sharing and the system is implemented. In the end, the authors compared transmitting full point cloud data and only dynamic objects from the viewpoint of throughput and latency. The results of experiments show that sharing full point cloud data is not realistic under IEEE 802.11ad communications. However, 700–900 Mbps was measured in the lab, which shows the potential of millimeter-wave communications. In [18,19], a proof-of-concept of cooperative perception using millimeter-wave communications was shown by sharing raw LiDAR sensor data. At the measurement part, the authors showed that approximately 900 Mbps was achieved.

The requirements for cooperative perception are actively studied by many groups. The 3rd Generation Partnership Project (3GPP) has published V2X (vehicle-to-everything) service requirements which include use cases for low-level to high-level automated driving [6]. In the case of an extended sensor service that is similar to cooperative perception service, 1 Gbps is required for high-level automated driving to prevent imminent collisions. Moreover, in the case of collective perception under raw sensor data transmission, 1 Gbps is required to visualize an all-around view [20]. On the other hand, 5G Automotive Association (5GAA), that develops end-to-end solutions for future mobility and transportation services, defines multiple groups based on 3GPP works, and presents requirements in multiple use case scenarios for C-V2X (cellular-V2X) [21,22,23]. For example, cooperative perception corresponds to a use case of high-definition sensor data sharing that belongs to the group of autonomous driving. However, in high-definition sensor sharing, a specific data rate is not required.

In this paper, we focus on realizing cooperative perception by sharing raw sensor data and utilizing cooperative perception for safe automated driving. Although sharing raw sensor data indeed gives a heavy channel load, it is necessary to guarantee distributed verification, which will be useful in emergency cases such as an infrastructure system error. Moreover, sharing raw sensor data can contribute to liability problems in the case of accidents and improving the accuracy of object localization [20]. In [24], it discusses sharing raw and processed sensor data from the viewpoint of sensor fusion. High-level fusion, which shares the results of detection and tracking algorithm carried out by each sensor, can be realized under lower complexity and requires few communication resources. However, the process will cut off a part of the information in the raw sensor data. On the other hand, low-level fusion, which shares raw sensor data, can retain sensor information so that it has the potential to improve localization. Moreover, it can reduce the latency caused by the process and helps to improve the performance of time-critical applications. However, it requires large amounts of computational resources and communication resources and needs precise calibration among sensors to fuse their data.

By using millimeter-wave communications for sharing raw sensor data that provide large amounts of communication resources, one of the challenges in sharing raw sensor data can be solved. Therefore, the combination of sharing raw sensor data and millimeter-wave communications has a great synergy that can share raw sensor data without information loss and waiting time for the process.

In order to derive how much data rate is required to realize safe automated driving by sharing raw sensor data, we analyzed the minimum required data rate for safe automated driving. In [8], the overtaking scenario at a two-lane road is assumed and the required data rate is derived by considering the driving path of overtaking and the recognition process based on using feature points. In [9], a safe crossing scenario at an intersection is assumed. However, using edge points for recognition is too primitive so that it is not used in practical recognition processes.

In order to support sharing raw sensor data, we focus on millimeter-wave communications. As shown in the above works, by comparing the theoretical maximum throughput, 6.75 Gbps of IEEE 802.11ad, there is room for improving this throughput in off-the-shelf devices of IEEE 802.11ad. Firstly, we will derive the requirements for safe crossing by analyzing the relation between the sensor data rate and recognition range. In this derivation, we used more practical feature points than edge points. Then, expecting the potential of millimeter-wave communications, we will compare and discuss the realized safe crossing, which shows the power of millimeter-wave communication to support safe crossing.

## 3. Required Data Rate on V2I for Safe Crossing

### 3.1. Cooperative Perception and Intersection Scenario Description

Firstly, we introduce current traffic accidents and show the motivation of analyzing an intersection scenario for deriving the required wireless communication performance for cooperative perception. In Japanese statistics, traffic accidents are classified by road shape and type of accident [25]. Classifying by road shape, a two-lane roads are where the most traffic accidents take place in Japan. Therefore, in the previous work, this type of traffic accident is analyzed for safe automated driving [8]. Unsignalized intersections are where the second-largest amount of accidents occurs in Japan—accounting for 25%. In America, traffic fatalities involving unsignalized intersection account for 18% [26]. From these statistics, we focused on traffic accidents occurring at an unsignalized intersection. Moreover, it is shown in Japanese statistics that traffic accidents between vehicles account for 90% at an unsignalized intersection. Therefore, we focused on traffic accidents between vehicles that occurred at an unsignalized intersection.

In the case of automated driving vehicles, intersection managers are planned to prevent traffic accidents. The intersection manager can be separated into several factors such as V2X interfaces, conflict detection, and vehicle dynamics [27]. There two architectures for V2X interfaces, i.e., centralized and decentralized approaches. The main advantage of decentralized approaches is that infrastructures are not required, so they can thus be easily scaled and used in uncrowded intersections. On the other hand, centralized approaches follow a server–client scheme. Although the reliability of centralized approaches deeply depends on infrastructures, these approaches can reduce network overheads due to their centralized information. In this analysis, an intersection manager is not explicitly considered, but a simple intersection manager based on the geographical location of the vehicle is assumed to establish a connection of V2I communications, which can be implemented by centralized or decentralized approaches. Therefore, the control of the vehicle’s behavior such as velocity is performed by the vehicle itself. For example, in the proof-of-concept, we adopted dynamic network management based on the geographical location of a vehicle [18]. Since this analysis focuses on the relation between the performance of wireless communications and safe automated driving, we assumed the same simple communication rule as in the aforementioned work.

For vehicle movements, vehicle dynamics and conflict detection are related to intersection management. Models of vehicle dynamics can be classified into three models. The difference among these models is the dimension of vehicle movement and whether the surrounding environment such as road slope is considered. To detect a conflict or collision, grid maps or predefined paths are used. In grid maps, the location of a vehicle in each time step is expressed as a grid, and a conflict or collision occurs when two vehicles occupy the same grid at the same time. On the other hand, the expected paths are used to check conflict in predefined paths. In this analysis, the conflict decision was based upon comparing the predefined paths and the vehicle dynamics on the path is described as a one-dimension model as follows:

$$\begin{array}{c} \dot{x}=v\\ \dot{v}=a \end{array}$$

where *x* and *v* are the longitudinal position and velocity of the vehicle and *a* is the acceleration input to the vehicle. Considering the fact that there are works about speed estimation based on only LiDAR sensors, we assume that LiDAR sensors can obtain vehicle velocity [28].

The traffic environment at the intersection is also an important factor. This is because it takes a long time to change all driving vehicles to automated driving vehicles. Therefore, a mixture environment in which both human and automated driving vehicles exist at the intersection should be considered. Related works about managing intersections under the mixture traffic environment can be classified into signalized and unsignalized intersection models. In the case of signalized intersections, basically traffic lights and the intersection manager cooperate on preventing collision at the intersection. In [29], the management of a signalized intersection was analyzed and traffic lights at the intersection are controlled by a connected vehicle center. The control was based on the information obtained from traffic detection devices such as radar or LiDAR sensors on roadside units.

On the other hand, in [30,31], decision making for automated driving at an unsignalized intersection was analyzed by processing sensor data and the decision making experiment was performed. In [31], the authors analyzed safe merging at an unsignalized intersection by using probabilistic functions. Both works considered not only the mixture traffic environment but also the incomplete installation of V2X communications, i.e., all vehicles cannot necessarily use V2X communications.

Since our analysis focuses on the contribution of vehicle-to-infrastructure (V2I) communications to safe automated driving, we assumed that an RSU is set at the intersection and can always send sensor data to the ego vehicle. Namely, the ego vehicle that starts to enter the intersection can always receive the cooperative perception service. Cooperative perception shares sensor data obtained from different locations and perspectives in the driving environment so that blind spots can be visualized. Using the received sensor data, the ego vehicle tries to pass through an unsignalized intersection under the mixture traffic environment. However, there are no managers that send control messages to automated driving vehicles.

**Figure 1:**
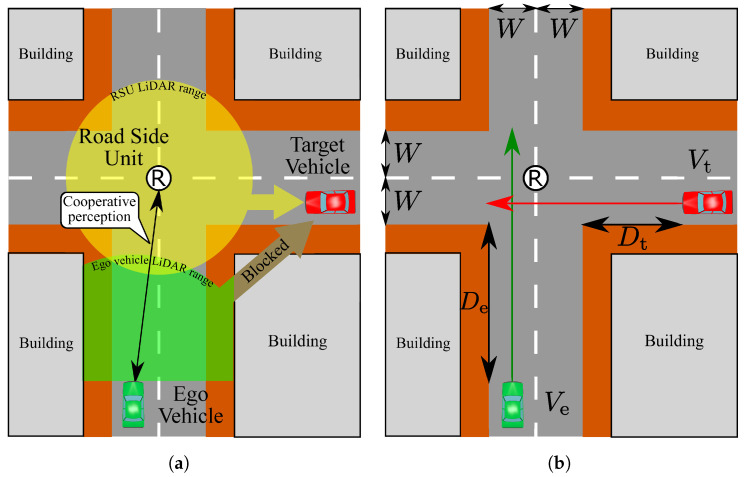
Overview of LiDAR sensors and the driving pattern at the unsignalized intersection: (**a**) The location and the range of LiDAR sensors. (**b**) The driving pattern of both ego and target vehicles.

From the above discussion, we focus on safe passing through an unsignalized intersection in the presence of a human driving vehicle. Figure 1 shows the assumed intersection scenario. The green ego vehicle is an automated driving vehicle and the red vehicle is a human driving vehicle. The goal of the ego vehicle is to pass safely through the intersection. However, the red vehicle also tries to pass through the intersection at the same time, which will lead to a collision at the intersection. From this assumption, the red vehicle becomes an important recognition target so that we call the red vehicle a target vehicle. For simplicity, the velocity of both vehicles is assumed to be constant. Since the target vehicle is a human-driven vehicle and there are no intersection managers, the ego vehicle has to recognize the target vehicle and decide whether a safely crossing is possible by itself. In order to accomplish this goal, an RSU is located at the intersection and can be used for cooperative perception. Namely, the ego vehicle receives LiDAR sensor data from the RSU that it can use for the recognition process with the sensor data obtained from the ego vehicle. Since the ego vehicle can know the location of the RSU by dynamic maps, we assume that the sensor data received from the RSU is automatically transformed into the ego vehicle coordinate. When the ego vehicle successfully recognizes the target vehicle, one of the simplest responses is always applying the brakes even if the collision does not occur at the intersection. However, this simple response will lead to traffic congestion at the intersection. Therefore, the ego vehicle should identify whether the collision will occur and apply the brakes in the case of collision, which can alleviate traffic congestion.

### 3.2. Vehicle Movement on Intersection

**Figure 2:**
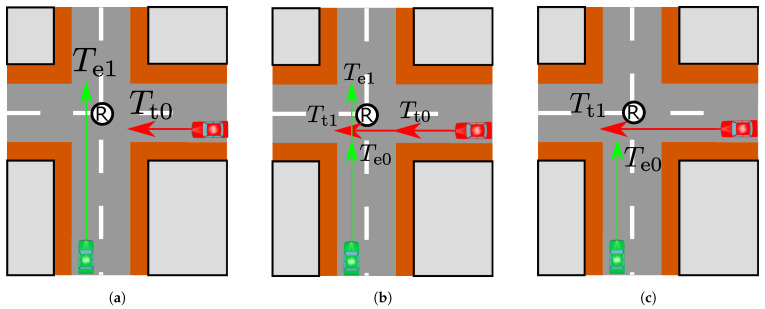
The three classified driving patterns: (**a**) The driving pattern in which the ego vehicle crosses first. (**b**) The driving pattern in which both vehicles are about pass through the intersection. and (**c**) The driving pattern in which the target vehicle crosses first.

In order to realize a safe and efficient crossing, the emergency cases where braking is necessary to prevent the collision should be specified in advance [32]. Moreover, this analysis helps to determine a required recognition range to prevent a traffic accident. Since driving at constant velocity is assumed, we can specify the collision cases by considering an arrival time and a leaving time at the intersection that depend on the initial positions of the vehicles. Therefore, we classified the collision cases and the other cases by these time parameters as Figure 2. Figure 2a (Figure 2c) is the driving pattern that the ego (target) vehicle first passes through the intersection. $T_{e0}$ ($T_{t0}$) is the time for the ego (target) vehicle to arrive at the intersection. $T_{e1}$ ($T_{t1}$) is the time for the ego (target) vehicle to leave the intersection. In the case of Figure 2a (Figure 2c), the relation of the time parameters is $T_{e1}\leq T_{t0}$ ($T_{t1}\leq T_{e0}$). On the other hand, Figure 2b represents the driving pattern through which the collision occurs and the relations of time parameters are $T_{t0}\leq T_{e1}\wedge T_{e0}\leq T_{t1}$. From the above classification, braking is only needed in the Figure 2b case.

From the assumption of constant velocity driving, these time parameters can be described by $V_{e},\;V_{t},\;D_{e},\;D_{t}$ and the relations of the time parameters in the collision cases can be described as follows:

(1) $$\begin{array}{c} \frac{D_{t}}{V_{t}}\leq \frac{D_{e}}{V_{e}}+\frac{2W}{V_{e}} \end{array}$$

(2) $$\begin{array}{c} \frac{D_{e}}{V_{e}}\leq \frac{D_{t}}{V_{t}}+\frac{2W}{V_{t}} \end{array}$$

where *W* is the width of the road as shown in Figure 1. Adopting comfortable braking to stop in front of the intersection, the following relation is used for simplifying Equations (1) and (2) [33,34]:

(3) $$\begin{array}{c} D_{e}^{\mathrm{brake}}=0.039\times \frac{V_{e}^{2}}{3.4} \end{array}$$

**Figure 3:**
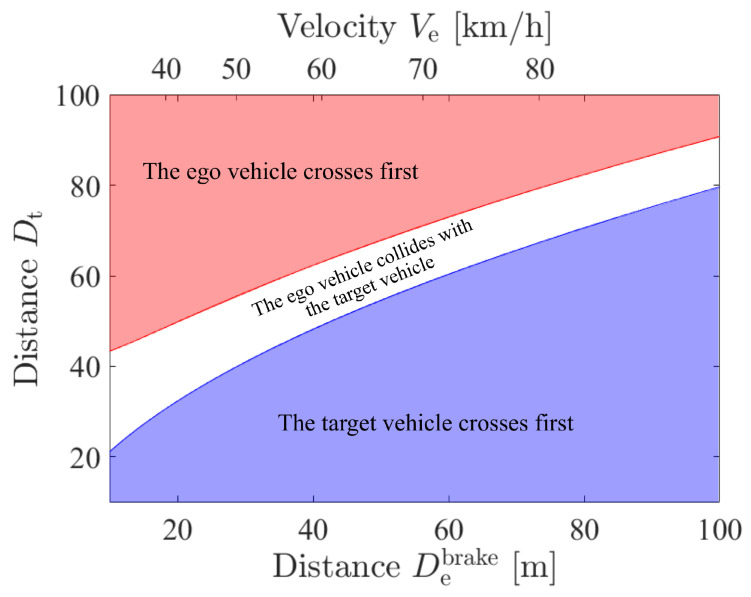
The visualized three driving patterns at $V_{t}=80$ km/h with comfortable braking.

Using the simplified inequalities, the three driving patterns with comfortable braking under $V_{t}=80$ km/h can be visualized, as shown in Figure 3. Notice that $D_{e}$ and $V_{e}$ have the relation of Equation (3), which are shown by the $D_{e}^{\mathrm{brake}}$ axis and $V_{e}$ axis in the figure. Since $D_{e}$ is related to both the braking distance and the performance of wireless communications, we use superscript to show the main role of $D_{e}$ in each analysis. Figure 3 shows the relation between the driving patterns and the initial locations of the ego vehicle and the target vehicle under the fixed velocity of the target vehicle. Namely, when the ego vehicle placed at $D_{e}^{\mathrm{brake}}$ starts to drive at the corresponding $V_{e}$, the decision making of the ego vehicle depends on the location and velocity of the target vehicle. When the initial location $D_{t}$ obtains very small (large) under fixed $V_{t}$, the target vehicle (the ego vehicle) passes through the intersection first so that it does not have to apply the brakes, which corresponds to the blue (red) area. On the other hand, when the distance and the velocity parameters of the ego and the target vehicle meet Equations (1) and (2), the collision occurs at the intersection, which corresponds to the white area. Therefore, when the parameters of the ego and the target vehicle belong to the white area, the ego vehicle has to perform comfortable braking to prevent the collision and stop in front of the intersection.

In order to perform the braking only at the collision cases, the ego vehicle has to recognize the target vehicle not in all cases but in the collision cases. This means that at least the ego vehicle has to recognize the target vehicle that is on the upper boundary of the white area. Therefore, the required recognition range $D_{t}^{\mathrm{req}}$ becomes the upper boundary of the white area and depends on $D_{e},\;V_{t}$, as shown in the figure. From the above discussion, substituting Equation (3) for Equation (1), $D_{t}^{\mathrm{req}}$ for comfortable braking is obtained as follows:

(4) $$\begin{array}{c} D_{t}^{\mathrm{req}}=V_{t}\sqrt{\frac{0.039D_{e}}{3.4}}+2W\cdot V_{t}\sqrt{\frac{0.039}{3.4D_{e}}} \end{array}$$

As introduced before, a constant velocity is assumed in this scenario, but there are many types of velocity scenarios of the ego vehicle such as driving with acceleration or deceleration. In this paragraph, we will discuss this topic. When $V_{e}$, $D_{e}$, and $V_{t}$ are given, driving with acceleration (deceleration) makes $T_{e0}$ in Figure 2b small (large) so that the required recognition range becomes short (long). Although a short recognition range requires a smaller sensor data rate than a long recognition range, entering into the intersection with acceleration is dangerous. On the other hand, driving with deceleration is safer than acceleration, but the required recognition range becomes large, which will become further away from tight requirements. Since we focus on not only safe automated driving but also tight requirements, we chose driving at a constant velocity which can be regarded as the average performance of driving with acceleration and deceleration.

### 3.3. Object Recognition Using CVFH

Since the ego vehicle has to recognize the target vehicle to decide whether comfortable braking is needed, a recognition process in the ego vehicle is necessary. In general, there are two ways to recognize an object, i.e., specific object recognition and general object recognition. In [8], specific object recognition using edge points was performed to recognize a vehicle. Since the recognition target is only a vehicle in this analysis, which is the same as in the above works, a specific object recognition was adopted. However, a more practical feature point than an edge point was used for recognition, and this improvement will provide a tight data rate requirement. In this recognition process, the clustered viewpoint feature histogram (CVFH), which is one of the global feature descriptors, is adopted and we use CVFH functions implemented in the Point Cloud Library (PCL) [35]. The reason for this adoption is that CVFH is robust to occlusions that often occur due to vehicles or buildings [36].

**Figure 4:**
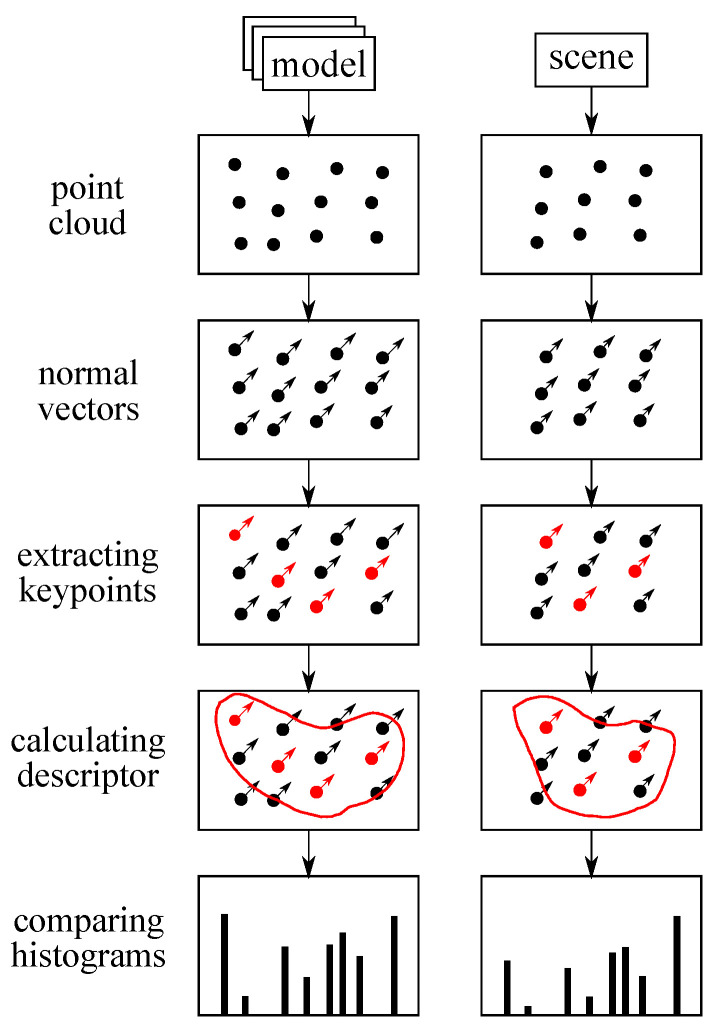
The process flow of object recognition.

A process flow of vehicle recognition is shown in Figure 4. This object recognition process consists of four processes and these processes are performed to both model and scene point cloud data. Since we adopt model base recognition, the model point cloud data are prepared for the object recognition process. In this recognition, the ego vehicle does not only recognize whether the obtained point cloud data are from a vehicle but also recognize the direction of the vehicle. The driving direction is specified for how the vehicle drives on a road and this check helps to guarantee that the traffic environment matches one of the classified driving patterns. The model point cloud data are generated by sensing a 3D vehicle model and a rectangular model under no obstacles in the assumed traffic environment. This data generation provides the maximum information for each model from the ego vehicle under the fixed locations of the vehicles. The rectangular model was prepared to check whether the ego vehicle has enough point cloud data to recognize the object and prevent fortunate recognition. On the other hand, the scene point cloud data are obtained by LiDAR sensors in the assumed traffic environment.

The object recognition starts from preparing a clustered point cloud. In this analysis, all points obtained by ray-trace simulation have a tag that tells which objects each point is on. By collecting points on the target vehicle, ideal clustering can be performed. The next process is the calculation of a normal vector for each point to calculate the CVFH. After preparing the clustered point cloud with normal vectors, keypoints are extracted from the clustered point cloud. In PCL, keypoints are explained as points that are stable, distinctive, and can be identified by using a well-defined detection criterion. The main advantage of using keypoints is that selecting points from the clustered points reduces the calculation time. Actually, instead of using keypoints modules, a voxel grid filter is often used to just reduce the number of points for convenience. The extracted keypoints were used to calculate CVFH and the calculation output histogram data. Finally, histograms calculated from the scene and the model point cloud are compared and the object and direction recognition is performed by choosing the nearest histogram. Since a large part of sensor data are obtained from the RSU, the histograms are made from the viewpoint of the RSU.

In order to choose the nearest histogram, the quantity that describes the similarity among histograms must be defined. There are several ways to compare histograms such as using correlation, chi-square distance, and intersection. Since chi-square distance is used to compare histograms generated by feature descriptors in the examples of PCL, chi-square distance is adopted as shown in the following equation:

(5) $$\begin{array}{c} d_{\mathrm{chi}}\left(H_{1},H_{2}\right)=\sum_{i}\frac{{\left(H_{1}\left(i\right)-H_{2}\left(i\right)\right)}^{2}}{H_{1}\left(i\right)+H_{2}\left(i\right)} \end{array}$$

The idea of a chi-square distance comes from regarding the difference between small bins as important.

**Figure 5:**
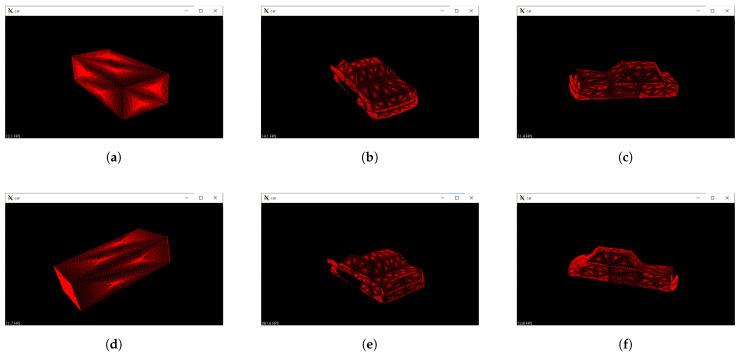
Examples of model point cloud data: (**a**) Model data of the front of the rectangular model. (**b**) Model data of the front of the vehicle. (**c**) Model data of the left of the vehicle. (**d**) Model data of the side of the rectangular model. (**e**) Model data of the rear of the vehicle. (**f**) Model data of the right of the vehicle.

There are six models which were prepared for recognizing the object and the direction by comparing the histograms. The visualized parts of the target vehicle are defined from the viewpoint of the ego vehicle. Figure 5 shows examples of compared point cloud data. As shown in the figures, there are two models of the rectangular model and four models of the vehicle model. The ego vehicle decides whether the object is the vehicle model or the rectangular model. When the ego vehicle does not have enough data, it recognizes the point cloud as the rectangular model. Moreover, the ego vehicle determines which direction the object faces among the four directions. Since the rectangular model has symmetries, there are only two models for the rectangular model.

**Figure 6:**
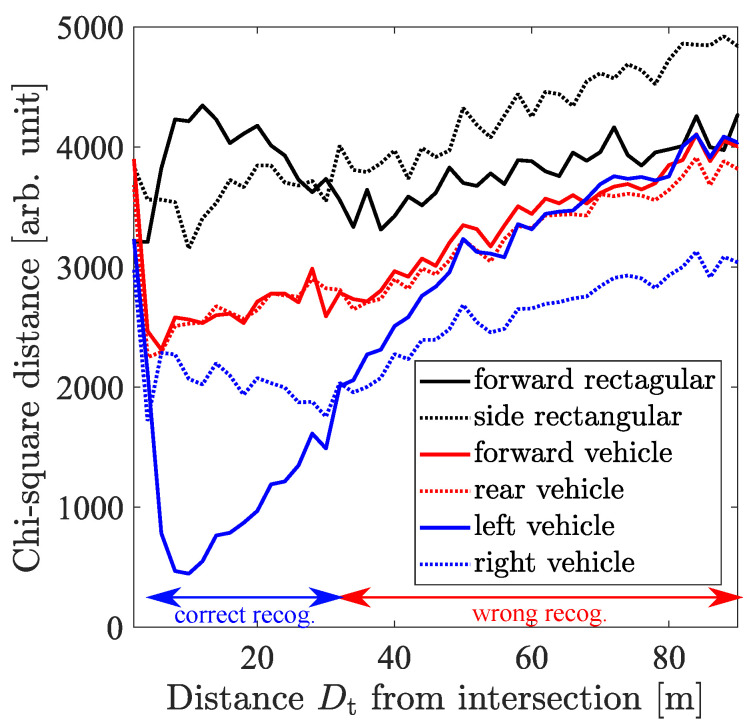
Transition of chi-square distance calculated by comparing CVFH histograms.

Figure 6 shows examples of the transition of chi-square distance between the model histogram and the obtained histogram. The transition of chi-square distance without cooperative perception is omitted because the buildings block almost all lasers from the LiDAR sensor on the ego vehicle to the target vehicle. In this scenario, when the ego vehicle recognizes the obtained point cloud as the left side of the vehicle shown in Figure 5c, the ego vehicle recognizes the correct traffic environment, which will lead to the correct braking decision. This is because all LiDAR sensor data are transformed into the ego vehicle coordinate as introduced in Section 3.1 and the left side of the target vehicle can be visualized from the ego vehicle under the intersection with no obstacles. From the figure, when the ego vehicle uses cooperative perception, it correctly recognizes the target vehicle in the range from $D_{t}=5$ to 32 m. Since the LiDAR sensor on the RSU does not see directly below the RSU, the recognition range does not start from 0 m.

One way to define the recognition range $d_{\mathrm{recog}}$ is choosing the maximum $D_{t}$ where the ego vehicle can correctly recognize the target vehicle. From Figure 6, when the wrong recognition range approximately 0 m is regarded as negligible, the maximum value is read as 32 m and the recognition range becomes 32 m. Although the maximum $D_{t}$ becomes the recognition range $d_{\mathrm{recog}}$ in this case, there are no guarantees that the ego vehicle can continuously recognize the target vehicle in general under this $d_{\mathrm{recog}}$ definition. Since the recognition range should guarantee the correct result within the range, the recognition range $d_{\mathrm{recog}}$ is defined as follows:

(6) $$\begin{array}{c} d_{\mathrm{recog}}\left(r_{\phi },\;r_{\theta }\right)=\max d_{0}\left(r_{\phi },\;r_{\theta }\right) \end{array}$$

(7) $$\begin{array}{c} s.t.\;\forall d<d_{0},\;\underset{m\in M}{\arg\;\min}\;d_{\mathrm{chi}}\left(H_{\mathrm{scene}}\left(r_{\phi },\;r_{\theta }\right),\;H_{m}|d_{\mathrm{recog}}^{\min}+d\right)=m_{l} \end{array}$$

where $m_{l}$ describes the left side of the vehicle model which is the correct model—as explained previously—$r_{\phi },\;r_{\theta }$ are the LiDAR sensor resolutions of the azimuth and elevation angle that are used to calculate point cloud and histograms.

### 3.4. Derivation of Required Data Rate

In order to derive the required sensor data rate $R_{\mathrm{req}}$, it is important to know how LiDAR sensors output sensor data. In this simulation, assumed LiDAR sensors scan the surrounding environment by spinning lasers at a certain frequency. Therefore, the scanning frequency, the number of points per scan, and the data size of one point give the sensor data rate of the LiDAR sensor. Considering the mechanism of the assumed LiDAR sensor, the required sensor data rate $R_{\mathrm{req}}$ to prevent the collision is formulated as follows:

(8) $$\begin{array}{c} R_{\mathrm{req}}=\left(\left\lfloor \frac{A_{\theta }}{\hat{r_{\theta }}}\right\rfloor +1\right)\times \left(\left\lfloor \frac{A_{\phi }}{\hat{r_{\phi }}}\right\rfloor +1\right)\times F_{\mathrm{scan}}\times D_{\mathrm{symbol}} \end{array}$$

(9) $$\begin{array}{c} \mathrm{where}\;\left\{\hat{r_{\phi }},\;\hat{r_{\theta }}\right\}=\underset{\left\{r_{\phi },\;r_{\theta }\right\}}{\arg\;\min}\;d_{\mathrm{recog}}\left(r_{\phi },\;r_{\theta }\right)>D_{t} \end{array}$$

where $A_{\phi }$ and $A_{\theta }$ are the scanning range in the azimuth and elevation angle, $F_{\mathrm{scan}}$ is the scan frequency (Hz) of the LiDAR sensor, and $D_{\mathrm{symbol}}$ is the amount of information per one laser point (bits).

By summarizing the analysis performed thus far, two relations are obtained. The first relation is between $D_{e}$ and $D_{t}$, which tells the recognition range $d_{\mathrm{recog}}$ required for the ego vehicle placed at $D_{e}$ to prevent the collision with the target vehicle driving at $V_{t}$. The second relation is between $d_{\mathrm{recog}}$ and the required sensor data rate $R_{\mathrm{req}}$, which tells the required sensor data rate $R_{\mathrm{req}}$ to realize the required recognition range $d_{\mathrm{recog}}$. Therefore, the sensor data rate required for the ego vehicle placed at $D_{e}$ can be obtained from the above two relations. However, in order to obtain the required sensor data rate for each $D_{e}$, many resolution sets of LiDAR sensors must be calculated. Therefore, a fitting curve is used to calculate the required sensor data rate for each $D_{e}$.

**Figure 7:**
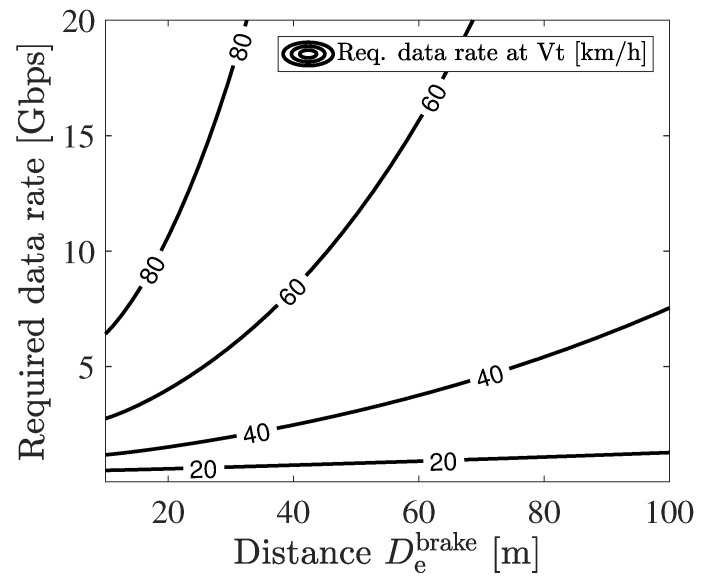
The sensor data rate required for the ego vehicle placed at $D_{e}$ to prevent a collision with the target vehicle driving at $V_{t}$.

Figure 7 shows the required sensor data rate to prevent a collision with the target vehicle driving at $V_{t}$ from the ego vehicle driving from $D_{e}$. From the figure, as the target vehicle velocity $V_{t}$ becomes high under a fixed $D_{e}$, the required sensor data rate becomes rapidly high. This is because the collision with the high-velocity target vehicle occurs in the case where the target vehicle drives from a distant place, i.e., a large $D_{t}$, which requires high-resolution LiDAR sensors to realize a long recognition range.

## 4. Performance Evaluation of Millimeter-Wave V2I

### 4.1. Millimeter-Wave V2I Communications

Since this analysis focuses on the relation between the performance of wireless communication and safe crossing, first of all, the current status of V2X communications is introduced in this section. As expected, wireless technology is a core factor of cooperative perception and the current candidates of wireless technology for cooperative perception are dedicated short-range communications (DSRC) and C-V2X. DSRC is natively designed to support vehicular networks so that it can communicate with high-mobility devices. However, the performance of DSRC rapidly degrades with high-density traffic. C-V2X makes a new channel that is called a sidelink channel for the absence of cellular infrastructure [37]. Sidelink channels enable vehicles to directly communicate through the PC5 interface and there are two modes that are called mode 3 and mode 4 in the sidelink channels. Mode 3 can be used in eNodeB (evolved node base station) coverage and eNodeB reserves resources. Mode 4 is made for the outside of eNodeB coverage and user equipment (UE) autonomously reserves resources. However, when traffic density increases, the performance of C-V2X degrades as that of DSRC [38]. In [39], the authors performed the experiment and compared the communication performance of IEEE 802.11p, which is one of DSRC and LTE-V, and showed that an RSU that transfers V2V messages improves the communication performance.

In order to meet the severe requirements of safe applications such as cooperative perception, evolved standards such as IEEE 802.11bd and NR-V2X are expected [40]. Since advanced PHY and MAC techniques are developed after the publication of IEEE 802.11p, the new evolved standard of IEEE 802.11p is expected for vehicular technology, which is called 802.11bd. On the other hand, NR-V2X is developed for severe requirements that are hard for C-V2X to fulfill. In [41], the PHY performance of IEEE 802.11bd and NR-V2X was evaluated, especially in terms of reliability. The results show that NR-V2X is superior to IEEE 802.11bd in terms of transmission reliability and mid-ambles significantly improve the performance of IEEE 802.11bd. The frequency bands for millimeter-wave communications are also actively discussed. Bands of 27, 37, 39, 60, 70, 80, and 90 GHz are all candidates for millimeter-wave communications. Although the 60 GHz band has oxygen absorption that severely limits its communication range, the 60 GHz band is attractive in terms of a global unlicensed band among these bands [42]. Therefore, we also assume using the 60 GHz band.

From the above introduction, we can see that there are many candidates for wireless communication systems. Therefore, we compared the cooperative perception for safe crossing realized by the candidates. The analysis was performed by comparing the sensor data rate and outage capacity calculated by the assumed channel model. In general, there are deterministic path loss models, statistical models, tapped delay line models, and geometry-based stochastic models to describe channel models for the 5.9 GHz frequency band [43]. In [44], the authors used a two-ray ground reflection model to compare V2I measurements in the 5.9 GHz frequency band. The results show that the model can properly represent the received power in LOS and NLOS environments. In [45], the authors analyzed millimeter-wave V2I communications and showed that a two-ray channel model well represents millimeter-wave communications with highly directional antennas. Considering the above works, we also assumed the V2I propagation model as a two-ray ground reflection model that is one of the deterministic path loss models, which will reduce calculation time.

**Figure 8:**
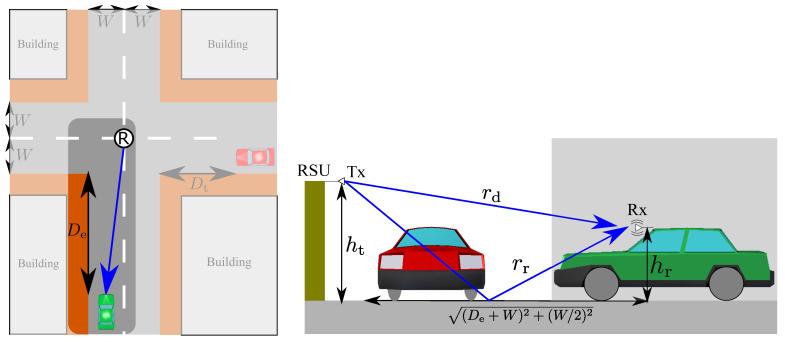
The two-ray ground reflection model with the receiver vibrating.

Figure 8 shows the assumed V2I propagation model. In this model, the receiver on the vehicle is vibrating due to a driving motor while the transmitter on the RSU is not vibrating [46]. Moreover, since automated driving vehicles can know the locations of RSUs by dynamic maps, the ideal beam alignment is assumed.

The received power is formulated as follows:

(10) $$\begin{array}{c} P_{r}=\frac{P_{t}}{L(r_{d})}{\left|\sqrt{G_{d}}\left(\frac{c}{4\pi f_{c}r_{d}}\right)+\sqrt{G_{r}}\left(\frac{c}{4\pi f_{c}r_{r}}\right)\Gamma e^{-j\{k\left(r_{d}-r_{r}\right)\}}\right|}^{2} \end{array}$$

where $P_{t}$ is the transmission power, $G_{d}$ and $G_{r}$ are the antenna gains for the direct and reflected wave, $r_{d}$ and $r_{r}$ are the optical path length for direct and reflected waves, $L(r_{d})$ is the absorption factor at 60 GHz by oxygen as 15 dB/km, *c* is the speed of light, $f_{c}$ is a carrier frequency, *k* is 2π/λ, and Γ is the complex reflection coefficient. A basic analysis of the effect of vibration on fading and height diversity has already been performed in a previous work [8]. Therefore, we focus on the effect of height diversity in the case of V2I communications.

**Figure 9:**
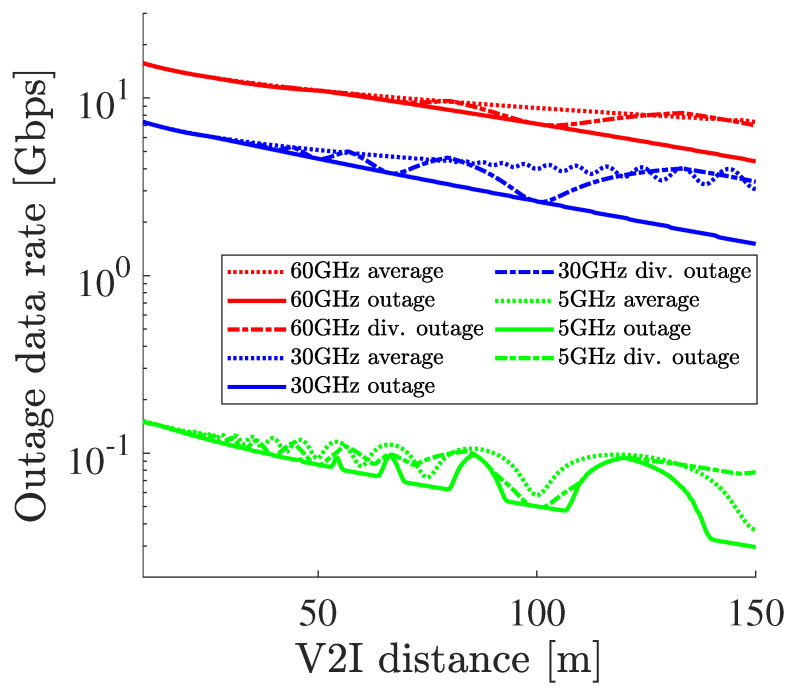
The 0.01% outage capacity with and without height diversity and the average capacity without height diversity.

Figure 9 shows 0.01% outage capacities using height diversity as well as not using height diversity and the average of channel capacity. Since the same parameters are used in the simulation, the parameters used in this calculation are summarized in the simulation section in Table 1. Here, 0.01% was based on the requirements for the reliability of transmitting raw sensor data published by 3GPP [20]. The 0.01% outage capacity was calculated by the following formula:

(11) $$\begin{array}{c} P(C\left(h_{r}|D_{e},\;f_{c}\right)<C_{\mathrm{out}}\left(h_{r}|D_{e},\;f_{c}\right))=0.01\% \end{array}$$

where the function P is the probability function about the capacity *C* of the V2I communication, and the capacity *C* and the outage capacity $C_{\mathrm{out}}$ stochastically change due to the vibrating receiver $h_{r}$ under the fixed $D_{e}$ and the carrier frequency $f_{c}$. From the figure, it was shown that height diversity certainly improves the outage capacity, but the increased amount of outage capacity is not drastically large.

This height diversity performance difference can be discussed from two aspects. The first aspect is the antenna vibration that causes a dynamic change in phase difference. Since no vibration at the receiver is assumed, a large phase difference between the direct path and the reflected path rarely occurs so that the improvement becomes smaller. The second aspect is the beamwidth of the antenna. Since millimeter-wave communications have a large path loss, its antenna needs strong directivity to realize long-range communications. In [46,47], a narrow beamwidth such as 10 degrees is used for the outdoor measurement of millimeter-wave communications. On the other hand, the narrow beamwidth was also adopted in our analysis to not only realize long-range communication but also utilize spatial diversity, or spatial channel reuse, in dense traffic. Using such a narrow beamwidth in V2I communication, the difference in the angle of departure between the direct path and the reflected path becomes large so that the reflected path does not depart from the high gain of the main lobe. Therefore, the effect of the destructive interference becomes small due to the small power of the reflected wave. However, the effect of the constructive interference also becomes small so that the outage capacity does not rapidly decrease.

### 4.2. Theoretical Speed Limitation

From the above discussion, the safe crossing can be formulated as follows:

$$\begin{array}{cc} \mathrm{safe}\;\mathrm{passing}: & V_{t}\leq V_{t}^{\max}\left(V_{e}^{\mathrm{safe}},\;f_{c}\right)\\ \; & \mathrm{where}\;V_{t}^{\max}=\max V_{t}^{\mathrm{safe}}\left(V_{e}^{\mathrm{safe}},\;f_{c}\right)\\ \mathrm{dangerous}\;\mathrm{passing}: & \mathrm{otherwise} \end{array}$$

where a pair of $\left\{V_{e}^{\mathrm{safe}},\;V_{t}^{\mathrm{safe}}\right\}$ is the velocity that ensures no collision at the intersection, and $V_{t}^{\max}$ is the maximum velocity of $V_{t}^{\mathrm{safe}}$ under the given $V_{e}^{\mathrm{safe}}$. The carrier frequency $f_{c}$ also relates to $V_{t}^{\max}$ because it relates to the recognition range that becomes the basis of the safe crossing. The details of $\left\{V_{e}^{\mathrm{safe}},\;V_{t}^{\mathrm{safe}}\right\}$ is shown as follows:

$$\begin{array}{c} \left\{V_{e}^{\mathrm{safe}},\;V_{t}^{\mathrm{safe}}\right\}\;s.t.\;C_{\mathrm{out}}\left(f_{c},\;D_{e}\left(V_{e}^{\mathrm{safe}}\right)\right)>R_{\mathrm{req}}\left(D_{e}\left(V_{e}^{\mathrm{safe}}\right),\;V_{t}^{\mathrm{safe}},\;D_{t}\right) \end{array}$$

From the assumption, $\left\{V_{e}^{\mathrm{safe}},\;V_{t}^{\mathrm{safe}}\right\}$ has to meet the relation that the outage capacity $C_{\mathrm{out}}$ is higher than the required sensor data rate $R_{\mathrm{req}}$ to prevent the collision by cooperative perception.

In our scenario, a single target vehicle was assumed thus far, but multiple target vehicles on the left and right lanes should also be discussed. Therefore, in this paragraph, we expanded to multiple target vehicles and discussed this scenario. When there are oncoming vehicles on the lane of the ego vehicle and an oncoming vehicle tries to turn right, the ego vehicle has to recognize it to prevent a collision, which means that the oncoming vehicle becomes a new target vehicle. However, for simplicity, we will focus on the case where target vehicles drive on the right and left lane. This is because oncoming vehicles are line-of-sight from the ego vehicle and recognition can be easier than this scenario. Firstly, the criterion for safe crossing should be formulated. The key factor is that the two inequalities for passing through the intersection in Equations (1) and (2) only depend on the distance and velocity. Therefore, the superposition of this criterion to all target vehicles ensures the safe crossing with no collision, and it is formulated as follows:

$$\begin{array}{cc} \mathrm{safe}\;\mathrm{passing}: & \forall V_{t}^{i}\in V_{t},\;s.t.\;V_{t}^{i}\leq V_{t}^{\max}\left(V_{e}^{\mathrm{safe}},\;f_{c}\right)\\ \mathrm{dangerous}\;\mathrm{passing}: & \mathrm{otherwise} \end{array}$$

where $V_{t}$ is the set of the velocity of all target vehicles, and $V_{t}^{i}$ is the velocity of the *i*th target. From this formula, we can use $V_{t}^{\max}$ the same way we used a single vehicle scenario. We regarded $V_{t}^{\max}$ as the speed limitation for the safe crossing.

### 4.3. Performance of Millimeter-Wave V2I Communications to Support Safe Crossing

**Figure 10:**
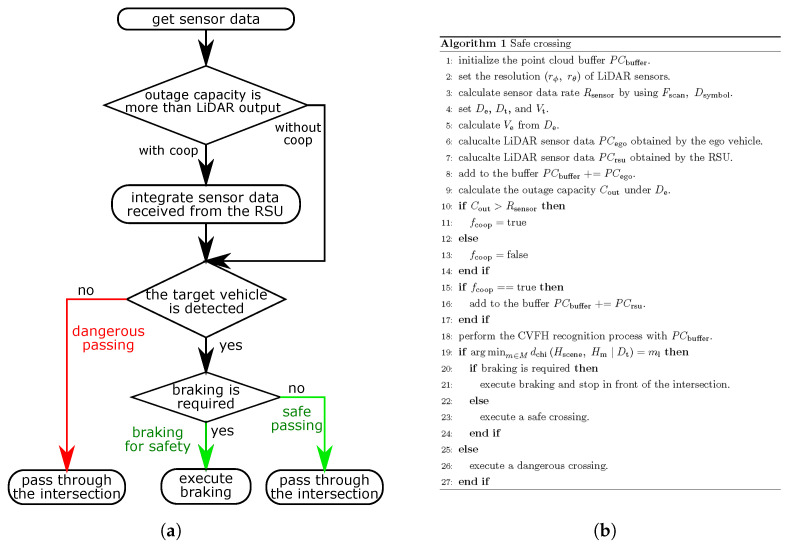
Description of the whole process in the simulation: (**a**) Block diagram of a safe crossing. (**b**) Algorithm of a safe crossing.

To estimate the safe crossing realized by conventional V2I communications and millimeter-wave communications, we performed the simulation. Figure 10a,b show the process flow of the simulation. This algorithm consists of a sharing sensor data part and recognition part. The sharing sensor data part is based on the previous work [8] and we checked that it works in the real environment in [18]. The recognition part is based on the typical model base recognition process of CVFH, which was also released in the robot operating system and used for object recognition in practice. Firstly, the parameters such as the resolution of the LiDAR sensors, the distance, and the velocity were set. Then, the LiDAR sensor data obtained from the ego vehicle and the RSU were simulated. Since this LiDAR sensor simulation is the same as the previous work, we omit this part and leave the explanation to the reference [8]. The LiDAR sensor model used in this simulation was based on Velodyne VLS-128, which can look downward deeper than upward. Since the RSU has to mainly sense downward, this model is appropriate for the intersection scenario. Under the assumed scenario and settings, a large part of lasers from the LiDAR sensor on the ego vehicle is blocked by buildings in many cases so that it does not provide more information than the LiDAR sensor on the RSU. Therefore, sensor data obtained from the ego vehicle were omitted to shorten the calculation time and sensor data obtained from the RSU which is mainly used. As previously assumed, the point cloud received from the RSU is transformed into the ego vehicle coordinate. Moreover, considering that LiDAR sensors can estimate the velocity of a vehicle, the ego vehicle can know the velocity of the target vehicle. After the LiDAR sensor simulation, the outage capacity between the RSU and the ego vehicle is calculated under $D_{e}$ to check whether the ego vehicle can use cooperative perception. To simplify the system, the ego vehicle can use cooperative perception when the outage capacity is more than the LiDAR sensor data rate. When the ego vehicle uses cooperative perception, it can only use its sensor data but also the sensor data of the RSU for the recognition process. Then, the recognition process based on CVFH was performed. When the recognition result corresponds to the target vehicle, the ego vehicle can decide whether comfortable braking is necessary to prevent the collision. Otherwise, the ego vehicle believes that no vehicles are entering the intersection, which will lead to a collision.

**Figure 11:**
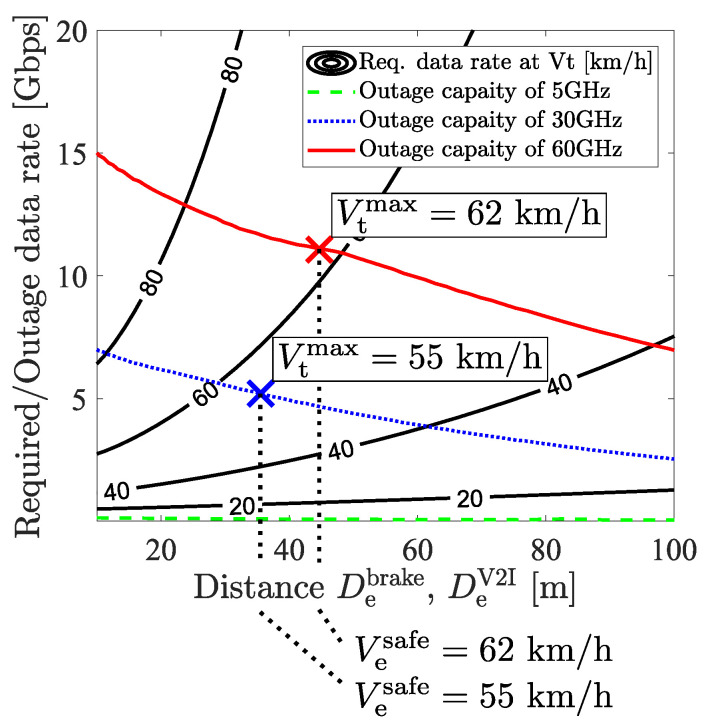
The contour plot shows the required sensor data rate at each $V_{t}$ that adopts comfortable braking and the color lines show the outage capacity realized by each carrier frequency.

Figure 11 shows the result of the simulation and Table 1 shows the parameters used in this simulation. The x axis describes $D_{e}$ which relates to the braking distance and the outage capacity of the V2I communication. The contour plot shows the required sensor data rate as same as the plot in Figure 7. The red, blue, and green lines show the realized outage capacity using 60, 30, and 5 GHz V2I communication.

The safe velocity pair $\left\{V_{e}^{\mathrm{safe}},\;V_{t}^{\mathrm{safe}}\right\}$ realized by each carrier frequency is described by the area below each red, blue, and green line. The velocity values are obtained by reading the contour plot and $D_{e}$. As $D_{e}$ becomes large, the decrease in the outage capacity and the increase in the required sensor data rate for each $V_{t}$ are read from the figure. The decrease in the outage capacity is expected to become far from the RSU, and the increase in the required sensor data rate is due to the necessity of a long recognition range.

In order to estimate the ability of cooperative perception at each carrier frequency, we focused upon the maximum velocity pair that both $V_{e}^{\mathrm{safe}}$ and $V_{t}^{\max}$ obtain the same velocity and obtain maximum the carrier frequency, which we will call the maximum safe velocity set. This estimation comes from reflecting two aspects. The first aspect is that the ego vehicle should pass through the intersection as quickly as possible to alleviate traffic congestion. The second aspect is that the ego vehicle also wants to prevent collision with the high-velocity target vehicle. By this estimation, it is shown that passing through the intersection with comfortable braking at 62 (55) km/h requires 11 (5) Gbps which can be supported by 60 (30) GHz. On the other hand, 5 GHz does not have the ability to send raw sensor data. From this result, we conclude that millimeter-wave V2I communications are needed to ensure safety at a realistic velocity and have better potential for safe driving than conventional V2I.

We will also discuss the recognition performance difference between edge point recognition used in the previous work and CVFH [9]. In the case of edge point recognition, since extracting edge points is performed by principal component analysis for each keypoint, the complexity becomes O(nk), where *n* is the number of keypoints and *k* is the number of neighbor points for each keypoint [48]. On the other hand, CVFH is the combination of Euclidean clustering and VFH calculation so that its complexity is near to O(nk). However, CVFH has to calculate Euclidean clustering additionally, and the preparation of the model data is more complex than that of edge points. Therefore, it is convenient to enable edge point recognition to realize the same recognition ability as CVFH by tuning the threshold from the viewpoint of reducing the calculation time. When the threshold is changed from 0.9 to 0.8 (0.77), the maximum safe velocity at 60 (30) GHz becomes the same result as CVFH.

## 5. Conclusions

In this paper, cooperative perception with raw sensor data is used to safely pass through the intersection and the required sensor data rate for the safe crossing is derived. Firstly, in order to reduce excessive braking, we specified the case where braking is required in the intersection scenario. Moreover, CVFH, which is a practical descriptor, was used to derive a more realistic required sensor data. From the result, it is shown that, as the velocity $V_{t}$ becomes higher, the required sensor generated data rate drastically increases. In the discussion part, it was shown that realizing cooperative perception by 30 and 60 GHz millimeter-wave communication has the ability to support safe crossing, while it is difficult for the conventional 5 GHz communications to support sharing raw sensor data to realize safe crossing. Since we want to estimate the ability of supporting the safe crossing, we focus on the maximum safe velocity set. The maximum safe velocity set shows that 30 and 60 GHz communications can prevent a collision with the target vehicle driving at approximately 60 km/h, which is a speed limit on a normal road in Japan. Finally, we compared CVFH recognition and edge point recognition. We found the appropriate threshold in the edge point recognition that realizes the same recognition ability as CVFH. This tuning will prevent excessively tight or loose recognition and edge point recognition can be used instead of CVFH so that the calculation time becomes short.

## Figures and Tables

**Table 1:** Simulation parameters.

LiDAR Parameters	
**Parameter**	**Value**
Location	Vehicle’s roof +20 cm
Range	200 m
Elevation Angle Range	−25° +15°
Elevation Angle Resolution $\left(r_{\phi }\right)$	$\begin{array}{c} {}[0.2{}^{\circ},\;0.1{}^{\circ},\;0.09{}^{\circ},\\ 0.08{}^{\circ},\;0.07{}^{\circ},\;0.06{}^{\circ},\\ 0.05{}^{\circ},\;0.04{}^{\circ},\;0.03{}^{\circ},\;0.02{}^{\circ}] \end{array}$
Azimuth Angle Range	360°
Azimuth Angle Resolution $\left(r_{\theta }\right)$	$\begin{array}{c} {}[0.2{}^{\circ},\;0.1{}^{\circ},\;0.09{}^{\circ},\\ 0.08{}^{\circ},\;0.07{}^{\circ},\;0.06{}^{\circ},\\ 0.05{}^{\circ},\;0.04{}^{\circ},\;0.03{}^{\circ},\;0.02{}^{\circ}] \end{array}$
Return Mode	Strongest
Scan Period	20 Hz
Data Size of One Point	16 bit (coordinate)
	+ 12 bit (power)
**V2I System Parameters in [5, 30, 60] GHz Bands**	
**Parameter**	**Value**
Height of Tx($h_{t}$)	5.0 m
Height of Rx($h_{r}$)	1.8 m
Transmitted Power	10 dBm
Boresight Gain	[4.3, 20, 26] dB
Antenna Aperture Size	2.6 cm × 2.6 cm
Polarization	vertical
Vertical Antenna Vibration Model	Gaussian (σ = 3.2 cm)
Bandwidth	[10, 500, 1000] MHz
Noise Figure	10 dB

## Data Availability

Not applicable.

## Abbreviations

The following abbreviations are used in this manuscript:

RSU	Roadside Unit
NHTSA	National Highway Traffic Safety Administration
CVFH	Clustered Viewpoint Feature Histogram
V2X	Vehicle-to-Everything
C-V2X	Cellular-V2X
3GPP	3rd Generation Partnership Project
5GAA	5G Automotive Association
ETSI	European Telecommunications Standards Institute
ITS	Intelligent Transport Systems Station
DENM	Decentralized Environmental Notification Message
CAM	Cooperative Awareness Message
CPM	Collective Perception Message
V2I	Vehicle-to-Infrastructure
PCL	Point Cloud Library
DSRC	Dedicated Short-Range Communications
UE	User Equipment
eNodeB	Evolved Node Base Station
V2V	Vehicle-to-Vehicle
